# Supplementary figures and images for: Prehemodialysis arteriovenous access creation is associated with better cardiovascular outcomes in patients receiving hemodialysis: a population-based cohort study
Source: PeerJ. 2019 Apr 3;7:e6680. doi: 10.7717/peerj.6680 (PMC6451437; doi:10.7717/peerj.6680)

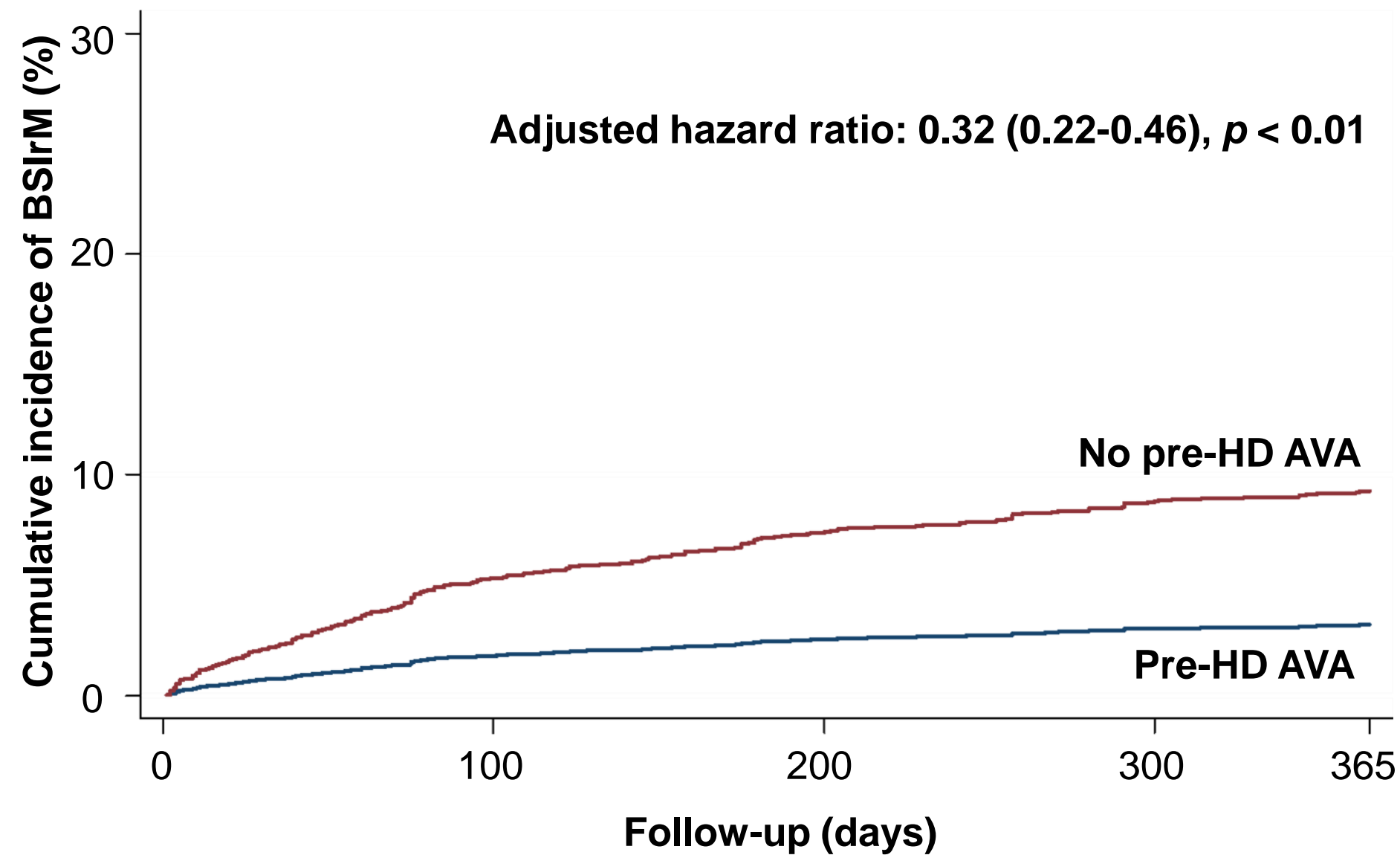

Supplement: Supplemental Information 1 — BSIrM, blood stream infection-related mortality; Pre-HD AVA, prehemodialysis arteriovenous access. [file peerj-07-6680-s001.pdf]
